# Long Short-Term Memory–Model Predictive Control Speed Prediction-Based Double Deep Q-Network Energy Management for Hybrid Electric Vehicle to Enhanced Fuel Economy

**DOI:** 10.3390/s25092784

**Published:** 2025-04-28

**Authors:** Haichao Liu, Hongliang Wang, Miao Yu, Yaolin Wang, Yang Luo

**Affiliations:** 1 School of Mechanical Engineering, North China University of Water Resourse and Electric Power, No. 36, Beihuan Road, Zhengzhou 450045, China; wx18937670329@163.com (H.W.); 18739032534@163.com (M.Y.); 17596513125@163.com (Y.W.); 2 Department of Mechanical Engineering, Faculty of Engineering, University of Malaya, Kuala Lumpur 50603, Malaysia; luoyang2925@163.com

**Keywords:** hybrid electric vehicle, LSTM speed prediction, DDQN, MPC, deep reinforcement learning

## Abstract

How to further improve the fuel economy and emission performance of hybrid vehicles through scientific and reasonable energy management strategies has become an urgent issue to be addressed at present. This paper proposes an energy management model based on speed prediction using Long Short-Term Memory (LSTM) neural networks. The initial learning rate and dropout probability of the LSTM speed prediction model are optimized using a Double Deep Q-Network (DDQN) algorithm. Furthermore, the LSTM speed prediction function is implemented within a Model Predictive Control (MPC) framework. A fuzzy logic-based driving mode recognition system classifies driving cycles and identifies real-time conditions. The fuzzy logic-based driving mode is used to divide the typical driving cycle into different driving modes, and the real-time driving modes are identified. The LSTM-MPC method achieves low RMSE across different prediction horizons. Using predicted power demand, battery SOC, and real-time power demand as inputs, the model implements MPC for real-time control. In our experiments, four prediction horizons (5 s, 10 s, 15 s, and 20 s) were set. The energy management strategy demonstrated optimal performance and the lowest fuel consumption at a 5 s horizon, with fuel usage at only 6.3220 L, saving 2.034 L compared to the rule-based strategy. Validation under the UDDS driving cycle revealed that the LSTM-MPC-DDQN strategy reduced fuel consumption by 0.2729 L compared to the rule-based approach and showed only a 0.0749 L difference from the DP strategy.

## 1. Introduction

### 1.1. Research Motivation

In the context of addressing global climate change, energy crises, and urban air pollution, hybrid electric vehicles (HEVs) have emerged as critical green transportation options, with significant practical implications for their study [1]. By combining internal combustion engines with electric motors, HEVs effectively reduce fuel consumption and greenhouse gas emissions, positioning themselves as strong alternatives to conventional internal combustion engine vehicles [2]. Compared to pure electric vehicles (EVs), hybrid electric vehiclesid electric vehicles are more feasible to implement in terms of technology and cost, particularly in areas lacking a comprehensive charging infrastructure or where long-term driving is often required. Consequently, further optimization of HEVs not only supports the adoption of clean energy in the transportation sector, but also provides an effective path to achieve the energy savings and emission reduction goals [3].

Research on energy management strategies for hybrid electric vehicles (HEVs) is fundamental due to its direct impact on fuel economy and emissions performance [4]. Energy management strategies optimize the real-time allocation of energy between the engine and the electric motor, allowing the vehicle to select the most efficient energy consumption mode in various driving conditions. Current studies indicate that advanced energy management approaches, such as strategies based on predictive control models and machine learning algorithms, can significantly improve energy efficiency and reduce emissions under actual operating conditions [5,6,7]. Furthermore, with the development of intelligent transportation systems, the integration of big data and traffic forecasting into HEV energy management allows predictive adjustments based on future road conditions, thus further improving fuel economy [8]. Therefore, research on HEVs and their energy management strategies aligns with global environmental protection trends and plays an irreplaceable role in promoting the development of sustainable transportation systems.

### 1.2. Literature Review

Currently, many countries are facing an energy crisis and environmental pollution issues, accelerating the transition from conventional fossil fuel vehicles to new energy vehicles (NEV) and driving unprecedented development in the NEV sector [9]. Compared to traditional internal combustion engine (ICE) vehicles, NEVs primarily include battery electric vehicles (BEVs), fuel cell vehicles (FCVs), and hybrid electric vehicles (HEVs). These vehicles show a significantly reduced dependence on fossil fuels or, in some cases, complete independence from them, making them more environmentally friendly [10]. In particular, plug-in hybrid electric vehicles (PHEVs) feature larger battery capacities than conventional hybrid vehicles, enabling them to meet the commuting needs of most office workers. As a result, PHEVs have garnered considerable attention and are regarded as one of the most promising research directions for the future.

Although battery electric vehicles (BEVs) produce nearly no environmental pollution, their driving range remains limited, and current technologies have yet to achieve parity with conventional gasoline-powered vehicles in terms of range. This limitation makes hybrid electric vehicles a viable compromise. In the future, with advances in battery energy storage technologies, BEVs are expected to completely replace traditional gasoline vehicles. To maximize the energy efficiency and sustainability of hybrid electric vehicles, it is essential to develop more effective energy management strategies (EMSs). By optimally distributing power between the engine and the battery, HEVs can operate within regions of high efficiency and high performance [11]. Research on energy management strategies can be broadly categorized into two main approaches: rule-based methods and optimization-based methods [12]. Furthermore, Ref. [13] classifies EMS into offline and online strategies.

Although various energy management strategies have been applied to PHEVs, the performance of existing methods in complex and dynamic environments remains insufficient. Traditional model-based control methods, such as MPC, often face challenges of high computational burden and poor real-time adaptability. While deep reinforcement learning (e.g., DDQN) has achieved success in other fields, its application in PHEV control still encounters issues such as high demand for training data and poor model stability. This paper proposes an integrated approach based on LSTM, MPC, and DDQN, aiming to enhance prediction accuracy through LSTM, optimize control decisions with MPC, and improve real-time learning capabilities using DDQN, thereby increasing the efficiency and adaptability of energy management. Through this innovative combined method, we can effectively address the issues of high computational complexity and poor real-time performance in existing methods, significantly improving system performance.

This paper focuses on the modeling and optimization of electric vehicle propulsion systems. The authors emphasize that the design of electric vehicle control systems must account for different dynamic characteristics, necessitating the establishment of mathematical models to describe these dynamics. For electric vehicles, the most critical dynamic characteristics include lateral dynamics (e.g., autonomous navigation) and longitudinal dynamics (e.g., energy efficiency and cruise control). This research direction has been extensively explored by numerous scholars and has evolved into a significant field of study. However, a pressing issue remains in the design of controllers based on longitudinal dynamic models: the parameterization of controller data. Since some parameters cannot be directly measured, this poses challenges for controller design and optimization. To address this issue, this paper proposes a motor dynamic testing method as an alternative to traditional torque sensor test benches. Experimental results demonstrate that by measuring the speed, current, and voltage of the motor during acceleration and deceleration, the torque and characteristics of the magnetic flux can be dynamically estimated more rapidly, conveniently, and accurately [14].

#### 1.2.1. Rule-Based Energy Management Strategy

Rule-based energy management strategies can be further categorized into deterministic rule-based strategies and fuzzy rule-based strategies. Deterministic rule-based strategies define a set of rules prior to implementation, specifying control parameter ranges or specific values, such as the state of charge (SOC) range of the battery, engine power, and motor torque. In Ref. [15], Anthony M. Phillips et al. successfully designed a controller based on a vehicle supervisory control (VSC) state machine. This controller employs a deterministic rule-based design that identifies all possible vehicle operating states and determines state transitions based on changes in driver demands and vehicle operating conditions. Fuzzy rule-based controllers, on the other hand, consist of rules established using fuzzy logic and are often combined with pattern recognition and neural networks. The primary advantage of fuzzy logic lies in its applicability to nonlinear time-varying systems, providing strong adaptability and ease of tuning. Additionally, fuzzy logic controllers can reduce computational burden. For instance, Hyeoun-Dong Lee and Seung-Ki Sul developed a fuzzy logic-based torque controller for parallel hybrid electric vehicles, which effectively reduced NOx emissions while ensuring vehicle driving performance and battery state-of-charge balance [16].

#### 1.2.2. Energy Management Strategies Based on Optimization

Offline global optimization requires prior knowledge of the entire driving cycle. Based on this information, methods such as dynamic programming (DP) and convex optimization are employed to solve the optimal power distribution problem. Lin et al. were the first to apply the DP strategy to enhance the energy management strategy of parallel hybrid electric trucks. They utilized dynamic programming techniques to determine the optimal power distribution of the hybrid system and subsequently formulated new rules to refine the control strategy, thereby improving fuel economy [17].

Although dynamic programming (DP) guarantees finding an optimal solution within a given range and can be easily implemented through coding, it suffers from a significant drawback—Bellman’s “curse of dimensionality” [18]. This limitation is particularly problematic in real-world vehicle operations, where driving information is often uncertain and conditions are highly dynamic. Due to the simplicity and practicality of DP, many researchers have attempted to enhance it through various approaches. For example, C. C. Lin et al. proposed an infinite-horizon stochastic dynamic optimization method that leverages the Markov process to reduce dependence on precise driving information [19]. However, offline global optimization algorithms are currently used primarily as benchmarks, with their solutions serving to improve online optimization algorithms.

Common online suboptimal control methods include the Charge Depleting–Charge Sustaining (CD-CS) strategy, the Equivalent Fuel Consumption Minimization Strategy (ECMS), and Model Predictive Control (MPC). Unlike offline global optimization, when applying online energy management strategies to plug-in hybrid electric vehicles (PHEVs), the dynamic variation in the battery’s state of charge (SOC) must be carefully considered. The CD-CS strategy has been widely adopted due to its simplicity, ease of implementation, and real-time capability [20]. However, it also has notable drawbacks, including the lack of optimality and the inability to efficiently manage battery power in real time.

Paganelli was the first to introduce the Equivalent Fuel Consumption Minimization Strategy (ECMS) into the energy management problem of hybrid electric vehicles. This algorithm is derived from the optimality conditions of Pontryagin’s Minimum Principle [21]. ECMS can achieve instantaneous optimal solutions without requiring prior knowledge of the driving cycle. Building on this, Musardo et al. proposed an adaptive ECMS that automatically adjusts the fuel equivalence factor through cycle prediction [22].

In the study of energy management strategies for electric vehicles in car-following scenarios, Chen et al. addressed the issue of model-based vehicle state prediction being affected by time-varying conditions. They proposed a data-driven Koopman Model Predictive Control (MPC) approach to optimize the speed of the following vehicles [23]. Experimental results demonstrated that the hierarchical predictive control using the Koopman model reduced energy consumption by 5.55% compared to hierarchical control based on mechanical models.

Sun et al. proposed a predictive energy management framework based on traffic data. Compared to conventional MPC, this framework introduces an additional SOC monitoring and planning layer, which rapidly generates battery SOC trajectories based on real-time traffic data to serve as the terminal state constraint for MPC [24]. Although MPC is widely recognized for its predictive capability and robustness, its optimization potential remains constrained by its dependence on accurate system models, which hinders further performance improvements [25].

#### 1.2.3. Predictive Energy Management and Artificial Intelligence Applications

In predictive energy management, the accuracy of speed prediction is closely linked to control performance, making the selection of an appropriate prediction horizon crucial for achieving better fuel economy. Within the MPC framework, the DP method is often employed to compute the optimal energy distribution at each MPC control step. In their study on MPC, Wang et al. found that as the prediction horizon increases, fuel consumption decreases, and the MPC strategy approaches the optimal solution [26]. For energy management strategies, the prediction horizon is also one of the most critical factors influencing optimal fuel economy.

In recent years, with the advancement of artificial intelligence, deep learning has been increasingly applied to energy management [27,28]. Wang, W. et al. proposed a vehicle speed prediction method that integrates a backpropagation (BP) neural network with a Markov chain. They combined speed prediction with the minimization of equivalent fuel consumption and used MPC to adaptively adjust the equivalence factor [29].

Xing et al. introduced a novel prediction approach that combines convolutional neural networks (CNNs) with Long Short-Term Memory (LSTM) networks [30]. Meanwhile, Ritter et al. proposed a stochastic MPC framework that incorporates long-horizon uncertainties into the energy management of hybrid electric vehicles (HEVs). This method integrates the optimal conditions of Pontryagin’s Minimum Principle with a scenario-based approach to achieve the computational efficiency required for real-time feasible energy management controllers [31].

#### 1.2.4. Mode Switching and Optimization of Hybrid Electric Vehicle

To address the challenge of employing different driving modes under varying driving conditions and to achieve more precise mode switching for improved fuel economy, Chen et al. proposed an Equivalent Fuel Consumption Minimization Strategy (ECMS) based on an intelligent dual neural network with Bayesian regularization. They introduced a novel equivalence factor correction method that adaptively adjusts the equivalence factor, enabling near-optimal fuel economy without relying on SOC reference [32].

Wei et al. designed a new driving mode recognition method that employs the K-means clustering algorithm to classify driving segments, thereby enhancing fuel economy and optimizing power distribution [33]. Similarly, Lin et al. developed an adaptive ECMS that leverages neural network techniques and a least squares method with a forgetting factor to predict vehicle speed and road grade. Their approach dynamically adjusts the equivalence factor based on the remaining distance in real time, leading to improved fuel efficiency [34].

Although these control strategies integrate neural networks with MPC and ECMS methods to predict future vehicle control and manage vehicle states, challenges remain in adjusting the equivalence factor within ECMS. Additionally, refining the prediction horizon in MPC remains an urgent issue to be addressed. Compared to MPC, reinforcement learning (RL) can be trained under various operating conditions to obtain more suitable parameters [35]. Yang et al. introduced RL into the MPC framework and combined it with a novel speed prediction model to develop a new energy management strategy, significantly improving fuel economy. However, despite leveraging reinforcement learning, this control strategy still lacks sufficient improvements in the speed prediction model [36].

#### 1.2.5. Contribution of This Paper

Previous rule-based control strategies have struggled to achieve better performance in complex environments, while neural-network-based reinforcement learning often focuses solely on current power demands, making it difficult to plan over unknown time horizons. Building upon previous research, this paper addresses the fuel economy issue of plug-in hybrid electric vehicles (PHEVs) with the following contributions:

A P2 architecture model for plug-in hybrid electric vehicles (PHEVs) is developed, including the vehicle’s drivetrain system, and the optimal operating curves for drivetrain components (engine and battery pack) are calculated.

A novel driving mode recognition system is designed, which uses a fuzzy logic controller to classify typical driving cycles into different modes and identify real-time driving modes.

An LSTM-based speed prediction model is developed, with a dataset that includes typical driving conditions such as FTP75, NEDC, and CBDC.

The DDQN algorithm is applied to optimize the initial learning rate and neuron dropout rate within the neural network, improving the accuracy of LSTM-based speed predictions.

In the MPC framework, energy management based on DDQN deep reinforcement learning is combined with speed prediction to control the output power of the drivetrain system, thereby enhancing the vehicle’s fuel economy.

Simulink simulations are conducted to compare the control performance of the LSTM-MPC-DDQN strategy with other strategies, and additional simulation experiments validate the real-time performance of the LSTM-MPC-DDQN control strategy.

This paper systematically investigates the optimization of energy management in plug-in hybrid electric vehicles (PHEVs). In Section 2, we comprehensively model the PHEV system, including the longitudinal dynamics model of the vehicle, the engine model, and the battery model, laying a solid theoretical foundation for the design of subsequent control strategies. Section 3 delves into the fundamental principles of Model Predictive Control (MPC) and integrates the Markov process in deep reinforcement learning with DQN and DDQN methods to construct an MPC-DDQN framework for more optimized control decision-making. Section 4 proposes a driving cycle decomposition method based on fuzzy neural networks, combined with LSTM for speed prediction, significantly enhancing the system’s prediction accuracy and environmental adaptability. Section 5 validates the effectiveness of the proposed method through simulation experiments, demonstrating significant improvements in computational complexity and real-time performance compared to traditional MPC methods. Section 6 summarizes the main contributions of this paper and provides an outlook on future research directions. The innovative method proposed in this paper offers an effective solution to the challenges of computational burden and real-time performance in PHEV energy management.

## 2. PHEV System Configuration and Modeling

**Figure 1:**
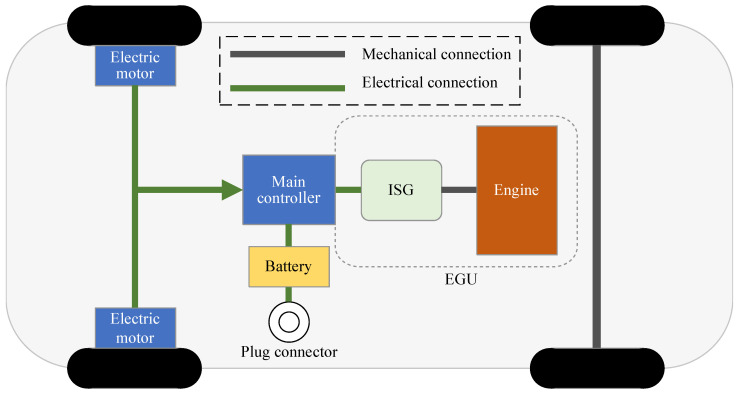
Plug-in hybrid vehicle structure.

The research subject of this paper is a plug-in hybrid electric vehicle (PHEV). The vehicle is powered jointly by an Engine Generator Unit (EGU) and a battery, where the EGU consists of an engine and an Integrated Starter Generator (ISG) motor that is mechanically connected. The battery is connected to the drive motor through a Main Controller. The schematic diagram of the Plug-In Hybrid Electric Vehicle used in this study is shown in Figure 1. The main component parameters are listed in Table 1.

**Figure 2:**
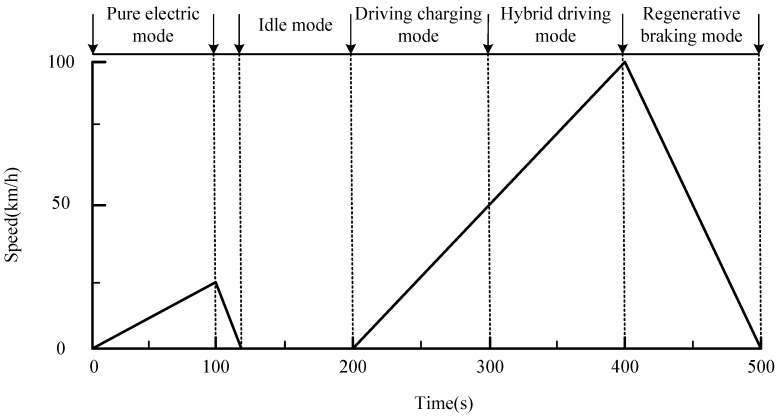
Driving mode.

**Figure 3:**
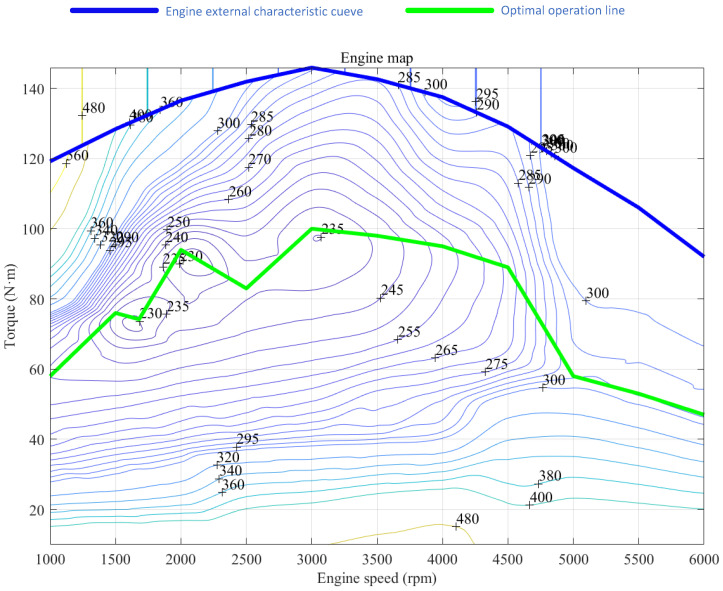
Engine map of the equivalent fuel consumption rate.

The operating modes of the plug-in hybrid electric vehicle (PHEV) are illustrated in Figure 2. These modes include five main operating conditions: pure electric vehicle mode, charge sustaining mode, hybrid drive mode, regenerative braking mode, and park mode. The criteria for determining the operating modes are shown in Figure 3.

PHEVs primarily operate in five modes: pure electric, charge while driving, hybrid drive, regenerative braking, and parking mode, as shown in Figure 2. The data of the five driving modes in Figure 2 are derived from Table 2.

In pure electric mode, the PHEV is driven solely by the electric motor, powered by the battery, and the internal combustion engine (ICE) remains off. This mode is typically used for low-speed or short-distance driving.

In driving charging mode, the ICE is the primary power source for driving the vehicle, while simultaneously charging the battery. This mode is usually activated during long-distance or high-speed driving to maintain or increase battery charge.

In hybrid driving mode, both the electric motor and the ICE provide power together. This mode is used when higher power output is required, such as during high-speed acceleration or uphill driving. The total power output is the sum of both sources.

During braking or deceleration, the PHEV’s electric motor operates in reverse to convert kinetic energy into electrical energy, which is stored in the battery. This improves energy efficiency by reducing waste.

In idle mode, the PHEV does not provide any power output, and both the ICE and electric motor are off. The vehicle is on standby, and the battery may only power auxiliary devices such as the air conditioning or multimedia systems.

$$\left\{\begin{array}{c} P_{EV}=U_{bat}\cdot I_{bat}\cdot \eta_{motor}\\ \left\{\begin{array}{c} P_{drive}=P_{engine}-P_{charge}\\ P_{charge}=U_{bat}\cdot I_{charge}\cdot \eta_{charge} \end{array}\right.\\ P_{HEV}=P_{engine}+P_{motor}\\ P_{regen}=\eta_{regen}\cdot \frac{1}{2}mu^{2}\\ P_{idle}=P_{auxiliary} \end{array}\right.$$

where $P_{EV}$ is the power output of the electric motor, $U_{bat}$ is the battery voltage, $I_{bat}$ is the battery current, and $\eta_{motor}$ is the motor efficiency.

where $P_{drive}$ is the driving power, $P_{engine}$ is the engine power output, and $P_{charge}$ is the charging power.

where $P_{HEV}$ is the total power, and $P_{motor}$ is the electric motor power.

where − is the regenerative power, $\eta_{regen}$ is the recovery efficiency, *m* is the vehicle mass, and *u* is the velocity.

where $P_{idle}$ is the power demand during idling, and $P_{auxiliary}$ is the consumption of auxiliary systems like air conditioning or entertainment devices.

### 2.1. Longitudinal Dynamics Model of the Vehicle

The vehicle model can be used to calculate the resistance encountered by the vehicle during its motion through longitudinal dynamics.

(1) $$F=G\cdot f\cdot \cos\alpha +\frac{C_{D}\cdot A}{21.15}\cdot u^{2}+G\cdot \sin\alpha +\delta m\frac{du}{dt}$$

where *G* is the vehicle’s gravity, N; α is the road’s slope; $C_{D}$ is the air resistance coefficient; *A* is the windward area, $m^{2}$; δ is the rotational mass conversion factor; *m* is the mass, kg; $\frac{du}{dt}$ is the driving acceleration, ${m/s}^{2}$.

### 2.2. Engine Model

In this paper, the efficiency distribution of the engine and generator is shown in Figure 1. The formula for calculating the fuel consumption of the engine is as follows:

(2) $$m_{f}=T_{eng}\times n_{eng}\times b_{e}\left(T_{eng},n_{eng}\right)$$

where $m_{f}$ is the fuel consumption rate, (kg/h); $T_{eng}$ is the engine torque, N·m; $n_{eng}$ is the engine speed, r/min; $b_{e}$ is the equivalent fuel consumption rate, kg/KWh.

In Figure 3, the blue line represents the external characteristic curve of the engine, while the green line indicates the optimal operating curve. The circular curve in the figure represents the equivalent fuel consumption rate, measured in units of kg/KWh.

### 2.3. Battery Model

The power battery is a crucial component of plug-in hybrid electric vehicles (PHEVs). There are two commonly used battery models: the Internal Resistance Model (Rint) and the Resistor–Capacitor Model (RC). In this paper, the Internal Resistance Model (Rint) is selected. The power battery is connected to the drive motor and generator through the Main Controller, supplying power to the drive motor or storing the electricity generated by the generator.

#### Rint Model

**Figure 4:**
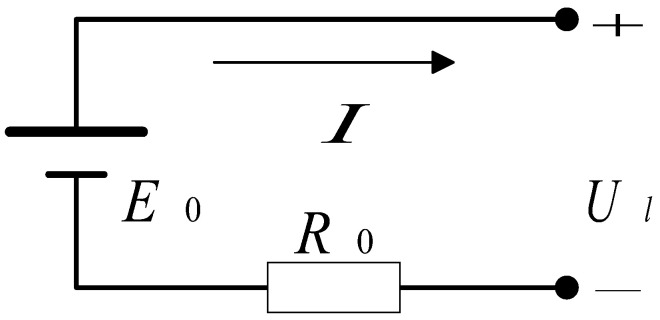
Schematic diagram of the internal resistance battery model.

The Internal Resistance Model treats the battery pack as an equivalent circuit consisting of an ideal voltage source in series with internal resistance, while the Resistor–Capacitor Model represents the battery pack as a circuit made up of two capacitors and three resistors. It is challenging to obtain accurate performance metrics for the battery solely through empirical formulas; thus, a more precise battery model can be developed by combining experimental data on the battery’s charge and discharge performance with empirical formulas. Therefore, the Internal Resistance Model is used to establish an equivalent model for the power battery, where the battery is represented as an ideal voltage source in series with a resistor, the Rint resistance model is shown in Figure 4.

For the internal resistance battery model, there are:

(3) $$U_{l}=E_{0}-IR_{0}$$

where $U_{l}$ is the load voltage; $E_{0}$ is the electromotive force (EMF) of the battery; *I* is the charge/discharge current; and $R_{0}$ is the internal resistance of the battery. The formula for calculating the battery output power, which is a function of current, is given by:

(4) $$P_{m}=IU$$

(5) $$I=\frac{E-\sqrt{E^{2}-4RP_{m}}}{2R}$$

where $P_{m}$ is the output power of the battery.

**Figure 5:**
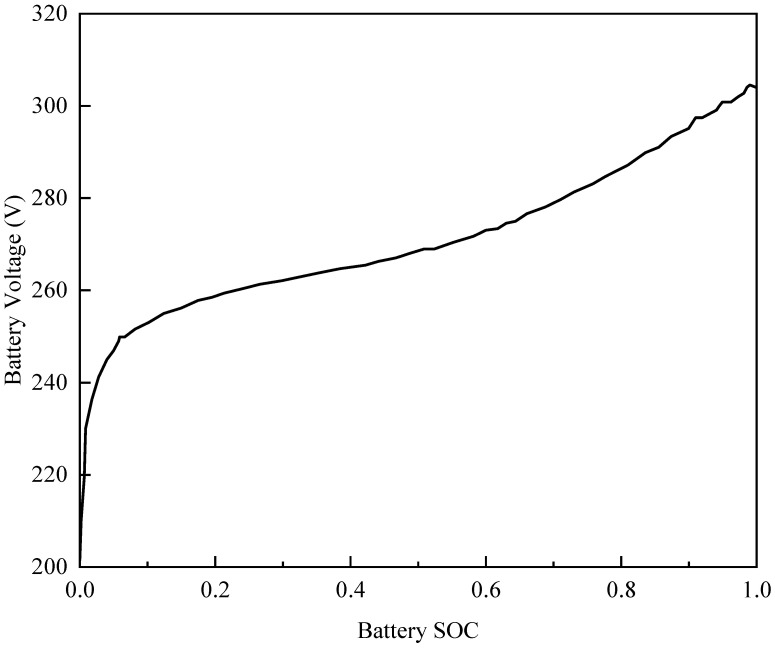
Graph of battery voltage variation with SOC.

The electromotive force (EMF) and internal resistance of the battery are influenced by both the temperature and the state of charge (SOC). This study neglects the impact of temperature on EMF and internal resistance, focusing primarily on the effect of SOC. For the calculation of battery SOC, the Ampere-Hour Integral Method is employed. The formula for calculating the SOC of the battery is given by:

(6) $$SOC=SOC_{0}-\frac{I}{Q_{b}}$$

where $SOC_{0}$ is the initial SOC value, and $Q_{b}$ is the battery capacity, the battery voltage variation with SOC as show in Figure 5.

By combining Equations (5) and (6), the charge and discharge efficiency of the battery pack can be expressed as:

(7) $$\eta_{dis}=\frac{P_{m}}{P_{b}}=\frac{2P_{m}R}{(E-\sqrt{E^{2}-4PR_{m}})E}$$

(8) $$\eta_{chr}=\frac{P_{b}}{P_{m}}=\frac{(E-\sqrt{E^{2}-4PR_{m}})E}{2P_{m}R}$$

where $\eta_{chr}$ is the charging efficiency of the battery; $\eta_{dis}$ is the discharging efficiency of the battery; and $P_{b}$ is the power of the battery, which is less than 0 during charging and greater than 0 during discharging.

## 3. Fundamentals of Model Predictive Control

### 3.1. Model Predictive Control

**Figure 6:**
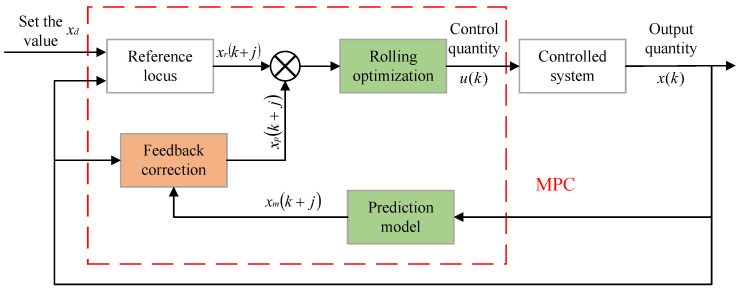
Schematic diagram of MPC structure.

This study integrates speed prediction and deep reinforcement learning energy management within the MPC framework. MPC is a flexible control framework that can be combined with any control algorithm to achieve real-time control. The fundamental idea of MPC is to utilize an existing model, the current state of the system, and future control variables to predict the future output of the system. The control process of MPC consists of four components: trajectory reference, prediction model, rolling optimization, and feedback correction. The solving process of MPC is illustrated in Figure 6.

Reference locus: The reference trajectory refers to the expected output or state trajectory $x_{r}$ of the optimization model. Through MPC, the output x(k) or state of the system is aimed to follow the reference trajectory $x_{r}$.

Prediction model: The prediction model is designed to forecast the dynamic behavior of the system over future time horizons. The prediction model can derive output information within the prediction horizon based on current or historical data using relevant prediction algorithms, transfer functions, and other methods, providing input information for the rolling optimization process.

**Figure 7:**
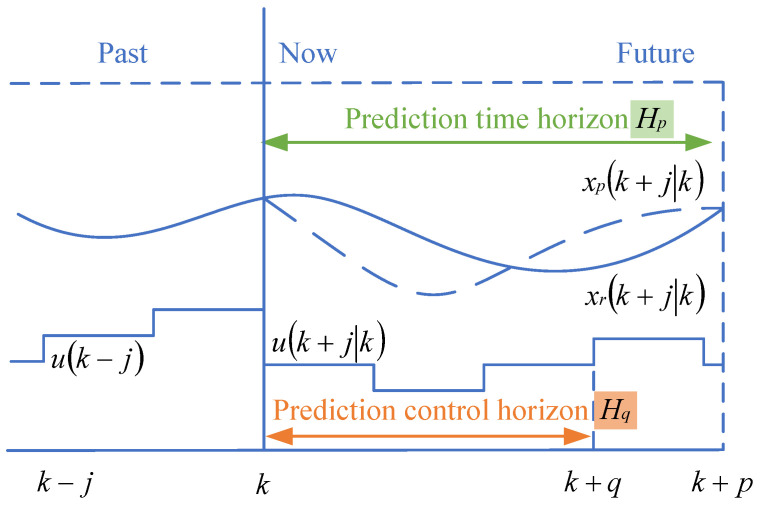
MPC principle diagram.

Rolling optimization: This is the most critical step. Rolling optimization is used to comprehensively consider the reference trajectory and the input information from the prediction model to solve the optimization problems within the prediction horizon. Different optimization problems will have corresponding solution methods, commonly including linear programming, dynamic programming, sequential quadratic programming, and fuzzy optimization. Compared to other optimization control algorithms, especially global optimization algorithms, the essential difference in the rolling optimization step of MPC is that its objective function is not static; rather, it is continuously updated based on changes in the reference trajectory. Therefore, the optimization objective function may vary in each prediction horizon, and rolling optimization aims for optimality within the rolling time frame. As shown in Figure 7, rolling optimization is not conducted just once. As the prediction horizon is updated, the optimization is repeatedly performed. At the time *k*, the rolling optimization process yields the optimal control sequence $\left[u\left(1|k\right),u\left(2|k\right),\ldots ,u\left(H_{p}|k\right)\right]$ within the prediction horizon, and the first control action u(1|k) is applied to the controlled system.

Feedback correction: In real-world environments, the controlled system is often affected by random disturbances and other factors. Additionally, changes in the operating environment may lead to model mismatch issues due to unmodeled factors, resulting in discrepancies between the predicted output of the prediction model and the actual state variables. Therefore, by introducing a feedback correction mechanism in a closed-loop system, the predicted output of the prediction model can be corrected in real time, reducing prediction errors and increasing the robustness of the system.

Regarding the feed-forward compensator, we have expanded the discussion to clarify its role in Model Predictive Control (MPC) for setpoint tracking. As you correctly pointed out, the feed-forward compensator utilizes trajectory preview information to adjust control inputs ahead of time, which can improve tracking performance. However, we emphasize that the effectiveness of the feed-forward term depends on how accurately the model can predict future trajectories and how well the system can handle the predicted control inputs.

We have also highlighted that combining feed-forward and feedback control in MPC can improve the robustness and accuracy of trajectory tracking. However, in cases of model mismatch or uncertainty, the benefits of feed-forward compensators can be limited. This article chooses to use the basic MPC without adding feedforward. The choice of the future time horizon is crucial for the integration of speed prediction and energy management. However, the results of speed prediction indicate that as the prediction horizon increases, the accuracy of speed predictions decreases. While a shorter prediction horizon offers higher prediction accuracy, it makes it difficult for the control strategy to plan effectively within a limited timeframe, which diminishes the advantages of prediction-based control strategies. Therefore, prediction horizons shorter than 5 s and longer than 20 s are not suitable for this study. Based on previous research, this study sets the prediction horizons at 5 s, 10 s, 15 s, and 20 s.

**Figure 8:**
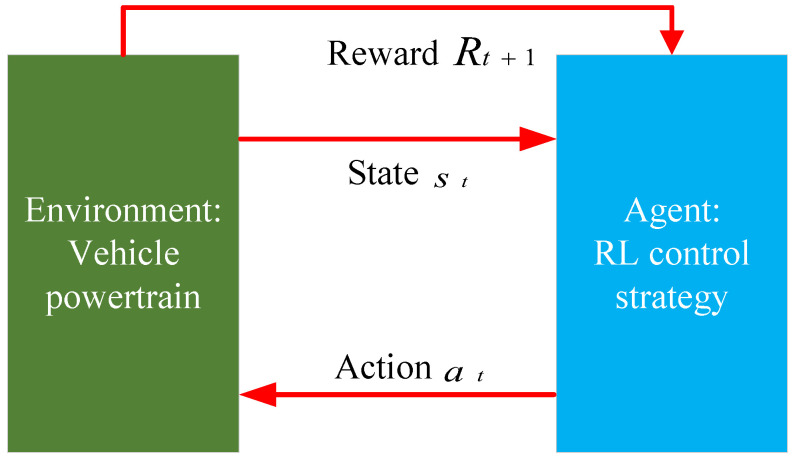
Reinforcement learning framework diagram.

Unlike supervised and unsupervised learning, reinforcement learning requires continuous interaction with the environment and adjusts strategies through trial and error. Reinforcement learning does not classify or label data based on existing training samples; instead, it determines the optimal action sequence through the ongoing interaction between the agent and the environment. The learning model of reinforcement learning is illustrated in the figure below. First, the agent obtains the state $s_{t}$ from the environment and takes action $a_{t}$ based on that state. Then, the environment updates the state $s_{t+1}$ as a result of the action $a_{t}$ and provides the agent with a reward $R_{t}+1$, which can be either positive or negative, based on the effects of the different actions. Through continuous interaction, the agent is trained to make better decisions. The energy management section of this paper focuses on the Double Deep Q-Network (DDQN) algorithm, the reinforcement learning framework diagram is shown in the Figure 8.

### 3.2. Morkov Process in Deep Reinforcement Learning

Deep reinforcement learning tasks are typically modeled as Markov Decision Processes (MDPs). By incorporating rewards, a Markov process is transformed into a Markov Reward Process, and subsequently, an action set is added to form the MDP. In an MDP, a quintuple (S,A,P,R,γ) is composed of a state set *S*, an action set *A*, a state transition probability matrix *P*, a reward function *R*, and a discount factor γ. In this study, we have constructed a deep reinforcement learning energy management model where the battery SOC and the predicted speed within the forecasting time horizon serve as the states, while the range extender output power is designated as the action. In reinforcement learning, both actions and state transitions are stochastic. Given a state *s*, the action is random. Denote the probability density of executing an action *a* in a state *s*; similarly, the state transition is also random given state *s*, with the state transition probability distribution represented as $P(s^{\prime }|s)$. During the state transition process, the environment generates different rewards *R* based on state changes, and at each sampling moment, the environment produces a reward *R* [37]. The current step’s action evaluation value, $G_{t}$, is discounted, which represents the weighted sum from step to the final step, as shown in Equation (9):

(9) $$G_{t}=R_{t+1}+\gamma R_{t+2}+\gamma^{2}R_{t+3}+\cdots =\sum_{k=0}^{\infty }\gamma^{k}R_{t+k+1}$$

where $G_{t}$ is the discounted reward, γ is the discount factor, γ∈[0,1], $R_{t+k+1}$ is the reward for the t+k+1 th step, which has less impact and less weight for the more distance time in the future.

Since the reward *R* is a random variable, the discounted return $G_{t}$ is also a random variable. Therefore, at time *t*, it is impossible to obtain a specific value $G_{t}$. Consequently, it is necessary to evaluate the current action and state of the environment by calculating the expected value $G_{t}$. The expected value of the random variable is used to obtain the value function, and Equation (12) represents the expression of the action-value function.

(10) $$Q_{\pi }\left(s_{t},a_{t}\right)=E\left[G_{t}|S_{t}=s_{t},A_{t}=a_{t}\right]$$

where $Q_{\pi }$ is the action-value function, which is the expectation of the discounted return, and is a representation of the value of the subsequent states of the current state.

From Equations (9) and (10), the Bellman expectation equation for the action-value function can be introduced, as shown in Equation (11):

(11) $$Q_{\pi }\left(s_{t},a_{t}\right)=E_{\pi }\left[G_{t}|S_{t}=s_{t},A_{t}=a_{t}\right]=s,A_{t}=a$$

*s* and *a* represent the determined state and action, respectively. To eliminate the influence of the policy function π and to optimally evaluate the action taken under the current state, it is necessary to identify the optimal action-value function, as shown in Equation (12).

(12) $$Q^{*}=\underset{\pi }{max}Q_{\pi }\left(s_{t},a_{t}\right)$$

At this point, the optimal action for the agent $a^{*}$ is:

(13) $$a^{*}=arg\underset{a}{max}Q_{\pi }\left(s,a\right)$$

### 3.3. DQN

Compared with traditional Q-learning, Deep Q-Networks (DQNs) do not use a table to query Q-values corresponding to actions and states. Instead, they utilize a neural network Q(s,a;w) to approximate the optimal $Q^{*}\left(s,a\right)$ [38]. By continuously updating the parameters ω, the neural network enables Q(s,a;w) to gradually converge to $Q^{*}\left(s,a\right)$. Unlike deep learning, reinforcement learning employs Temporal Difference (TD) algorithms to update network parameters. TD algorithms do not require the entire simulation process to be run completely; instead, they train the network through ongoing interaction. The TD algorithm primarily trains the model based on its estimate of Q-values and actual rewards, as shown in Equation (14), which is used to estimate the Q-values at the time step *t* and t+1 in DQN.

(14) $$Q\left(s_{t},a_{t};\omega \right)\approx r_{t}+\gamma Q\left(s_{t+1},a_{t+1};\omega \right)$$

where $Q(s_{t},a_{t};w)$ represents the Q-value estimated by the neural network at the current time step, while $Q(s_{t+1},a_{t+1};\omega )$ represents the Q-value at the next time step. $r_{t}$ is the reward received by the agent after taking an action at the current time step, and ω represents the parameters of the neural network.

$Q(s_{t},a_{t};w)$ is the estimate made at the time step *t*. By time step t+1, the reward $r_{t}$ becomes known, making the right-hand side of the equation closer to the actual evaluation. The right-hand side is defined as the Temporal Difference (TD) target $y_{t}$. During the solution process, DQN selects the action *a* that maximizes $Q(s_{t+1},a_{t+1};\omega )$ as the next action, as shown in Equation (15).

(15) $$y_{t}=r_{t}+\gamma Q\left(s_{t+1},a_{t+1};\omega \right)=r_{t}+\gamma \cdot \underset{a}{max}Q\left(s_{t+1},a;\omega_{t}\right)$$

To bring the output of DQN closer to the TD target, the loss is calculated as the squared difference between the two, as shown in Equation (16). The network parameters are then updated based on the loss.

(16) $$L=\frac{1}{2}{\left(Q\left(s_{t},a_{t};\omega \right)-y_{t}\right)}^{2}$$

During training, DQN often uses experience replay to prevent the waste of experience. Experience replay not only enables the reuse of experience, thus avoiding its waste, but also allows the decomposition of experience sequences to eliminate the correlation between adjacent sequences. Before training the network, the performance can be adjusted by tuning the capacity *n* of the experience buffer (replay buffer). In this paper, *n* is set to 50,000. After determining *n*, the agent randomly samples it for training.

### 3.4. DDQN

**Figure 9:**
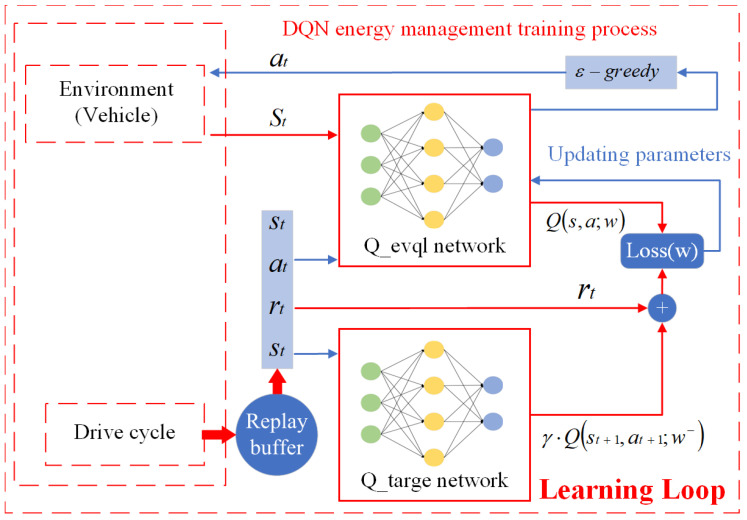
Double DQN training process.

In the process of estimating Q-values, DQN inevitably introduces errors. As shown in Equation (16), when calculating the TD target, a maximization operation is performed. However, due to the issue of bootstrapping, overestimation is inevitable. To mitigate the negative impact of this overestimation, the Double Deep Q-Network (DDQN) was proposed. DDQN uses two neural networks: $Q_{e}aval$ (the current value network) and $Q_{t}arget$ (the target value network). These networks share the same structure but differ in parameter updating methods: $Q_{e}aval$ updates its parameters with each training step, while $Q_{t}arget$ copies the parameters from $Q_{e}aval$ at longer intervals. As shown in Figure 9, DDQN first calculates the Q-value using the current network and computes the optimal action $a^{*}$ through a dynamic programming algorithm, as illustrated in Equation (17).

(17) $$a^{*}=arg\underset{a}{max}Q\left(s_{t+1},a;\omega \right)$$

Subsequently, the target network calculates the Q-value based on the optimal action $a^{*}$, as shown in Equation (18). Then, the TD algorithm calculates the loss value based on $y_{t}$ to update the current network.

(18) $$y_{t}=r_{t}+\gamma Q\left(s_{t+1},a^{*};\omega^{-}\right)$$

The loss value for DDQN is represented in Equation (19).

(19) $$L\left(\omega \right)=E_{(s,a,r,s_{t+1})∼D}\left[r_{t}+\gamma Q\left(s_{t}+1,a^{*};\omega^{-}\right)-Q{\left(s_{t},a_{t};\omega \right)}^{2}\right]$$

where *D* represents the experience replay buffer, γ is the discount factor, and $E_{(s,a,r,s_{t+1})∼D}$ denotes the expected loss sampled from the buffer *D*. The parameter update algorithm for the neural network employs the Root Mean Square Propagation (RMSProp) method. Compared to the Stochastic Gradient Descent with Momentum (SGDM) algorithm, RMSProp can adaptively adjust the learning rate. This method exhibits better adaptability to non-smooth objectives, reduces model oscillation, and is more conducive to stable convergence of the model.

### 3.5. MPC-DDQN

This paper uses an LSTM speed prediction model to forecast future speed over a time horizon and calculates the required power for the predicted time horizon. The currently required power, the future required power, and the current state of charge (SOC) are used as inputs to the state of the agent.

The agent is trained to generate the output power of the engine and battery pack for the next time step based on the state variables, with an output power range of [0 kW, 285 kW]. By adjusting the reward coefficient for each episode, the control strategy can make more optimal decisions. To reduce the training time cost, this paper separates the training of the speed prediction model from that of the energy management agent, using the prediction results as input to the Double Deep Q-Network (DDQN) environment. The pseudocode for the MPC-DDQN energy management is presented in Table 3.

In deep reinforcement learning, the design of the reward function is crucial as it directly affects the model’s convergence and learning efficiency. To ensure that the state of charge (SOC) remains stable around 30% while also saving fuel, the reward function in this paper is primarily composed of two components, as shown in Equation (20).

(20) $$r_{t}=\beta {\left(SOC\left(t\right)-SOC_{0}\right)}^{2}+\gamma \left(a_{e}qual\cdot \left(SOC\left(t\right)-SOC_{0}\right)+fe_{ins}\right)$$

where $r_{t}$ is the reward value at the current step; SOC(t) is the SOC at the current time; $SOC_{0}$ is the initial SOC; β and γ are two coefficients. After multiple simulations comparing control performance, the value of β is set to −160 to ensure that the reward function exhibits an increasing trend during the iterative process. Additionally, the parameter γ is set to 1.5; $a_{equal}$ is the equivalent conversion coefficient for fuel and electricity; and $fe_{ins}$ is the instantaneous fuel consumption.

### 3.6. Formulation of the MPC Framework

**Figure 10:**
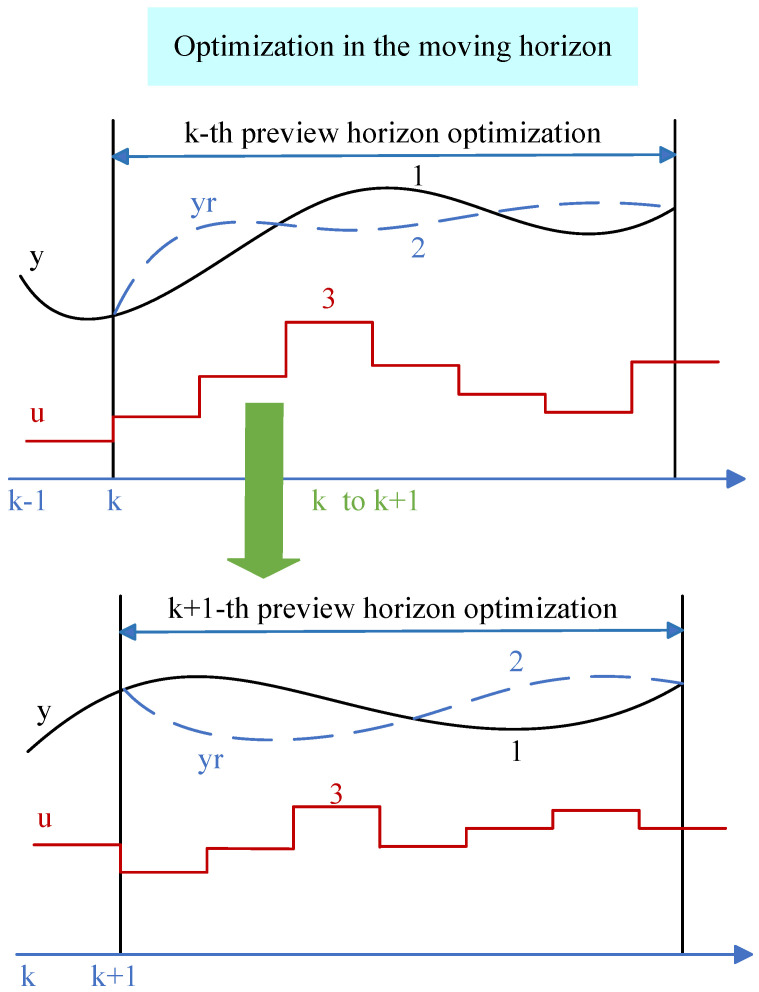
The schematic diagram of the MPC framework.

The MPC framework is developed for the real-time energy management of HEVs (Hybrid electric vehicles). The fundamental concept involves solving a constrained nonlinear optimization problem over a predictive horizon at each time step. After computing the optimal control sequence, only the first control input is applied to the powertrain system, while the process is repeated at the next step.The schematic diagram of the MPC framework is shown in Figure 10. According to Equation (21), the predictive model of the HEV is formulated as follows:

(21) $$\dot{x}=f\left(x,u,w\right),y=g\left(x,u,w\right)$$

where x=SOC is the state variable; $u={\left[\omega_{e},T_{e}\right]}^{T}$ is the control variable; $w=V_{predict}$ is the system disturbance; and $y={\left[SOC,{\dot{m}}_{f}\right]}^{T}$ is the output. Additionally, $V_{predict}$ is the future velocity sequence provided by the predictor.

The energy management of plug-in hybrid electric vehicles (PHEVs) aims to enhance performance metrics such as energy consumption efficiency or greenhouse gas emissions reduction. In the text, the objective function is defined to minimize the total cost over a specific driving cycle. The total cost includes the cost of energy consumption, the equivalent cost of battery degradation, and the equivalent social cost of carbon emissions. Based on this definition, the objective function is expressed as follows:

(22) $$J^{*}=min\left(J_{energy}+J_{bat}+J_{carbon}\right)$$

where $J^{*}$ is the total cost of the trip, $J_{energy}$, $J_{bat}$ and $J_{carbon}$ are the energy consumption cost, the equivalent battery life loss cost, and the social cost of equivalent carbon emission, respectively.

For plug-in hybrid electric vehicles (PHEVs), the energy consumption costs consist of both fuel consumption costs and electricity consumption costs. The calculation formula is as follows:

(23) $$J_{energy}=J_{fuel}+J_{elec}=\int_{0}^{T}\left[c_{f}m_{f}\left(t\right)+c_{e}m_{e}\left(t\right)\right]\;dt$$

where $c_{f}$ and $c_{e}$ are the unit price of the fossil fuel and electricity, respectively, $J_{fuel}$ and $J_{elec}$ are the cost of the natural gas consumption over the trip, *T* is the time length of the trip, and *t* is the time variable.

The fuel consumption rate $m_{f}\left(t\right)$ is calculated using the following equation:

(24) $$m_{f}\left(t\right)=\frac{b_{e}\left(t\right)P_{egu}\left(t\right)}{3600\rho_{f}}$$

where $P_{egu}\left(t\right)$ is the instantaneous output power of EGU, $b_{e}$(t) is the rate of fuel consumption of EGU, and $\rho_{f}$ is the density of natural gas.

The instantaneous electricity consumption $m_{e}\left(t\right)$ is calculated as follows:

(25) $$m_{e}\left(t\right)=\frac{P_{bat}\left(t\right)}{3600}$$

where the objective function can be further formulated as:

(26) $$J^{*}=min\int_{0}^{T}\left[c_{f}m_{f}\left(t\right)+c_{e}m_{e}\left(t\right)+\varphi \phi \frac{|I_{reat}\left(t\right)\Delta t|}{3600\Gamma_{nom}}+c_{c}m_{c}\right]\;dt$$

where φ is the purchase cost of the battery system and ϕ is the decay factor of the battery.

In this work, the discrete time step is 1 s. Additionally, the optimization problem is subjected to a set of inequality constraints:

(27) $$\left\{\begin{array}{c} \omega_{min}⩽\omega \left(t\right)⩽\omega_{min}\\ T_{min}⩽T\left(t\right)⩽T_{min}\\ I_{b,min}⩽I_{b}\left(t\right)⩽I_{b,max}\\ SOC_{min}⩽SOC\left(t\right)⩽SOC_{max} \end{array}\right.$$

where the subscripts (min and max) represent the minimum and maximum restriction. The solid black line indicates the reference trace, the dashed blue line indicates the optimal control output, and the red line indicates the optimal control input.

## 4. A Decomposition Method for Driving Cycles Based on Fuzzy Neural Networks

### 4.1. Driving Cycle Decomposition Approach

Traditional DPR-based power management methods tend to classify driving segments using known continuous driving cycles, and then develop control strategies by identifying the entire driving cycle. However, for a given driving cycle, these methods often include several types of driving segments that are easily overlooked. This is because different driving patterns can exhibit similar driving segments, and the same driving cycle can contain different types of segments. As a result, control strategies developed based on the entire driving cycle may struggle to ensure optimal vehicle performance. To overcome this limitation, a novel classification method is proposed—using a fuzzy neural network to classify driving segments, grouping them according to their characteristics.

In this study, we selected three typical driving cycles: FTP75, NEDC, and CBDC. For a given driving cycle, the number of parameters used to describe it can reach up to several types. However, too many parameters may significantly increase computation time and could potentially affect the accuracy of the results. In reference [39], the average speed is reported as the sole parameter. In our research, we use the average speed and maximum speed of each segment as the classification parameters. The methods for calculating average speed and maximum speed are as follows:

(28) $$V_{ai}=\int \frac{v}{t}\;dt$$

(29) $$V_{maxi}=max\left(V_{j},j=1,2,\ldots ,k\right)$$

where $V_{ai}$ represents the average speed of each driving segment, where *i* denotes the index of the driving segment, and $V_{maxi}$ refers to the maximum speed of each driving segment.

### 4.2. Fuzzy Neural Network Controller

Driving cycles are crucial for evaluating vehicle performance, especially when calculating fuel economy and emissions. Many standard driving cycles have been developed for different types of vehicles in various countries or scenarios. To better assess the performance of the energy management system (EMS), we designed a driving cycle controller based on a fuzzy neural network.

A fuzzy logic controller is used to classify driving segments and identify the driving types in the DPR process. The controller consists of four key components: fuzzification, rule base, fuzzy inference, and defuzzification. These components work together to effectively process and categorize the driving segments.

Fuzzification: We set the input variables of the fuzzification module to two parameters: average speed (km/h) and maximum speed (km/h), with the output module being the driving block pattern. For the pre-segmented driving blocks, the average speed and maximum speed are calculated accordingly.

**Figure 11:**
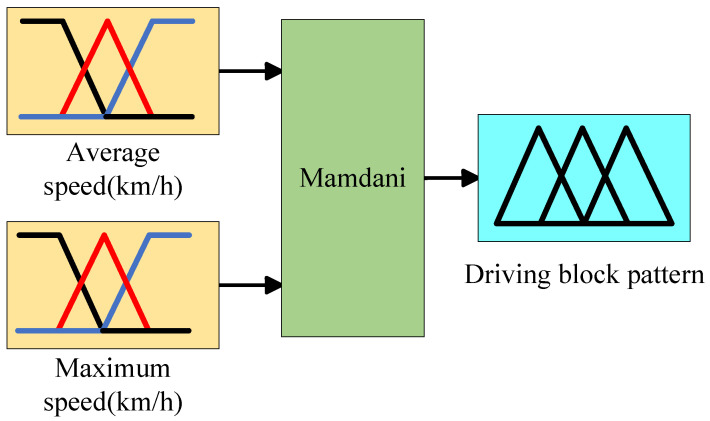
Fuzzy neural network controller.

As shown in Figure 11:

The linguistic terms for the input and output variables are set as Low-Level (low-speed driving pattern), Middle-Level (medium-speed driving pattern), and High-Level (high-speed driving pattern). The inference process utilizes Mamdani’s fuzzy theory. It is noteworthy that, in this study, the intervals corresponding to the three-speed patterns are shown in Table 4.

**Figure 12:**
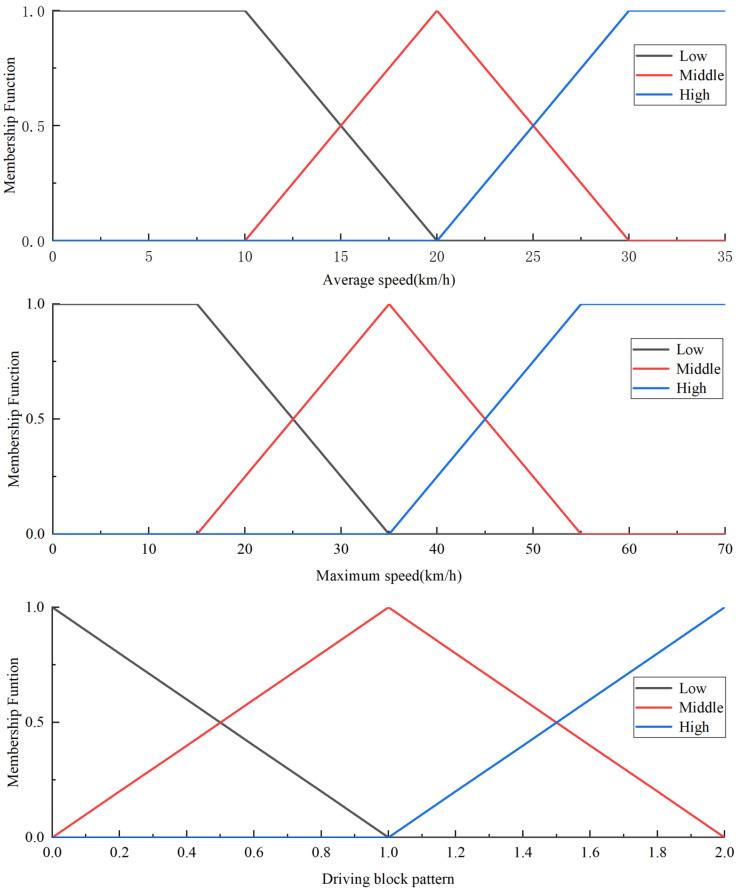
Membership function.

Based on the established rules, we can determine the membership functions for Lower-Level $\mu_{L}\left(\chi \right)$, Middle-Level $\mu_{L}\left(\chi \right)$, and High-Level $\mu_{L}\left(\chi \right)$. The membership function is shown in Figure 12.

Rule base: The fuzzy logic used in this study follows the A+B→C (If A and B, then C) pattern, where A represents the fuzzy set of average speed, which consists of three levels: $V_{a}-Low$, $V_{a}-Middle$, and $V_{a}-High$. B denotes the fuzzy set of maximum speed, which is also divided into three levels: $V_{max}-Low$, $V_{max}-Middle$, and $V_{max}-High$. C represents the fuzzy set of driving block patterns. The inference process is based on the Mamdani fuzzy theory. From each rule shown in Table 5, we can obtain the corresponding fuzzy relation matrix $R_{i}$ through the Cartesian product of $A_{i}$ and $B_{i}$. The overall fuzzy relation matrix *R* can be obtained by combining the fuzzy relations $R_{i}$ using the following equation:

(30) $$\left\{\begin{array}{c} R=R_{1}VR_{2}\ldots VR_{i}\ldots VR_{8}\\ R_{mn}=max\left(R_{1mn},R_{2mn},\ldots ,R_{imn},\ldots ,R_{8mn}\right) \end{array}\right.$$

where *m* and *n* (where m=1,2,3 and n=1,2,3 ) denote the indices of the matrix elements in the fuzzy relation matrix *R* and the fuzzy relation matrix $R_{i}$.

**Figure 13:**
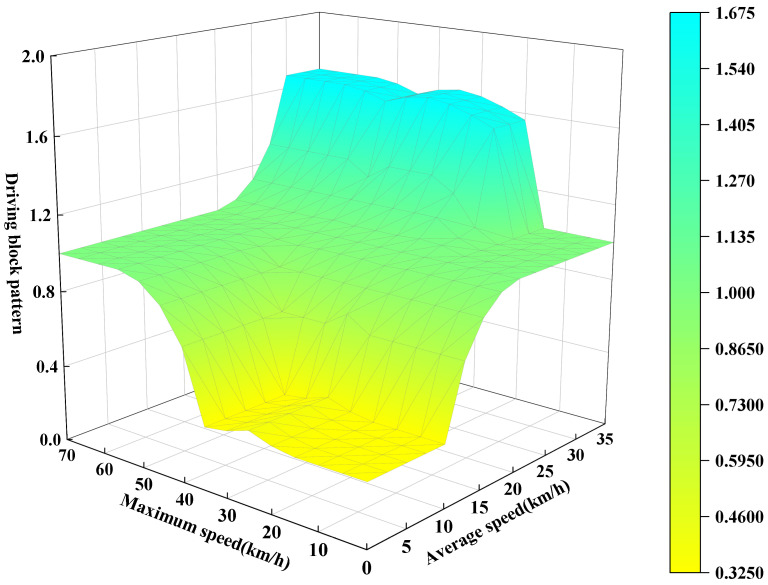
Three-dimensional coordinate graph of fuzzy rules.

Subsequently, we can examine the three-dimensional coordinate graph of the established fuzzy rules, as shown in Figure 13.

Fuzzy inference: When we input the fuzzy relation matrix *R* of the variables $A_{i}$ and $B_{i}$ and obtain the fuzzy results, we can use the following equation to determine the driving type:

(31) $$C_{i}=\left(A_{i}\times B_{i}\right)\cdot R$$

where $C_{i}$ represents the fuzzy set of the output variable.

Defuzzification: The result obtained through the fuzzy set formula $C_{i}=\left(A_{i}\times B_{i}\right)\cdot R$ is not applicable under real conditions. Therefore, we should convert it to a known driving pattern. The maximum membership principle is employed to determine the driving block pattern. According to this principle, the driving block pattern is identified at the value with the highest membership degree in the domain.

### 4.3. Driving Cycle Classification and Composition

**Figure 14:**
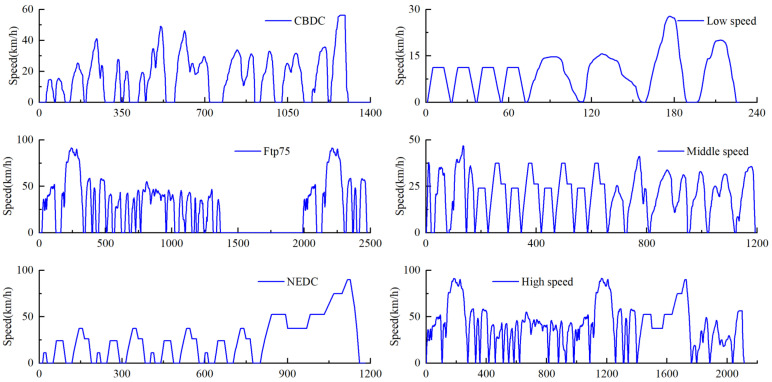
Classified driving cycles.

**Figure 15:**
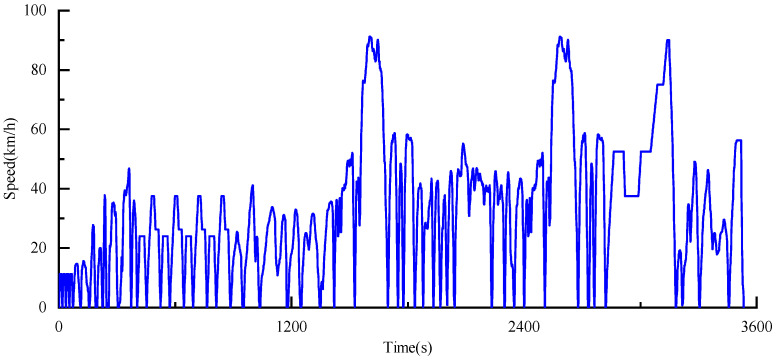
Speed composite conditions classified by neural network.

From Figure 14, we can observe that most driving blocks have similar profiles in the rearranged driving cycles. In the subsequent design, the composite conditions shown in Figure 15 will be used for the driving cycles.

**Figure 16:**
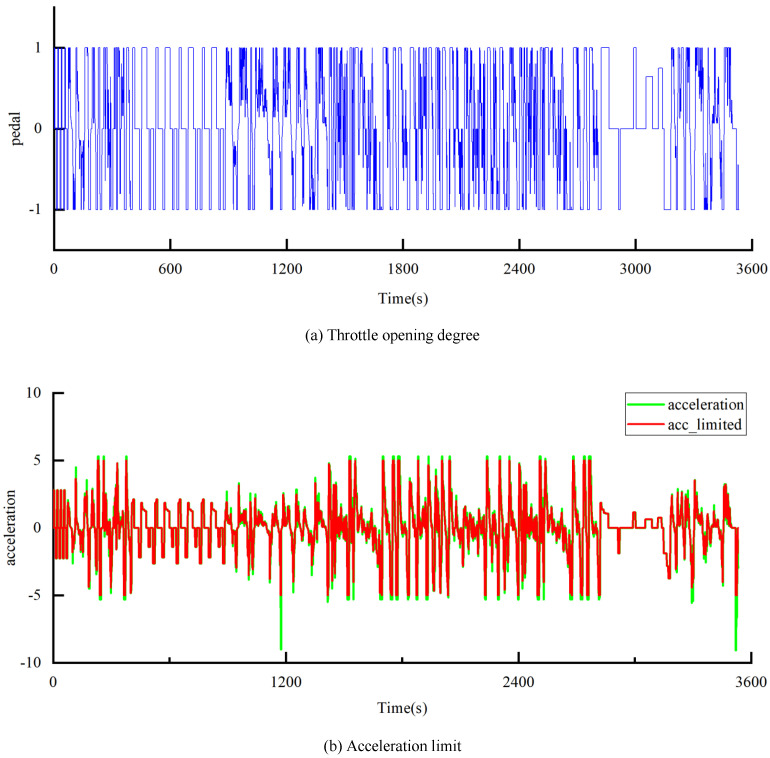
Throttle opening degree and acceleration limit.

Acceleration will be one of the reference criteria for the vehicle’s throttle opening degree. Figure 16a shows the throttle opening degree corresponding to the composite conditions. Figure 16b displays the acceleration data of the vehicle under the composite conditions. However, to ensure the authenticity of the driving conditions, this study sets the maximum acceleration boundary at 5 m/s^2^. The vehicle’s maximum braking speed is determined by the combination of mechanical braking, regenerative braking from the motor, and road conditions. Under normal driving conditions, considering passenger safety and ensuring the authenticity of driving conditions, the maximum braking speed limit is set to −5 m/s^2^, as indicated by the $acc_{limite}$ in Figure 16b.

### 4.4. Speed Prediction Based on LSTM

**Figure 17:**
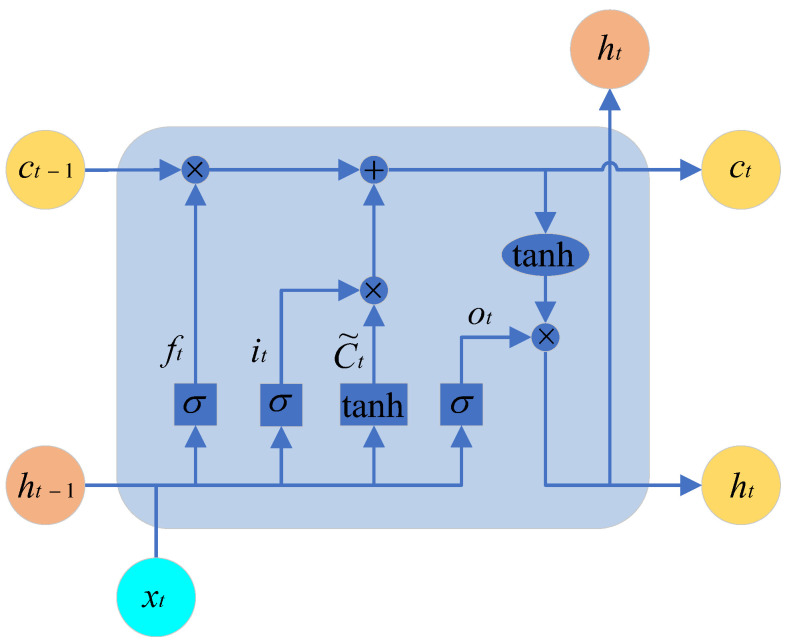
Schematic diagram of LSTM architecture.

LSTM (Long Short-Term Memory Network) is a commonly used deep learning model for processing sequential data. Compared to traditional RNNs (Recurrent Neural Networks), LSTM introduces three gates (input gate, forget gate, and output gate, as shown in Figure 17 and a cell state. These mechanisms enable LSTM to better handle long-term dependencies within sequences, with the gates represented by the sigmoid activation function.

Forget Gate: By operating on $x_{t}$ and $h_{t-1}$ and passing the result through the sigmoid function, we obtain a vector in the range of [0,1]. A value of 0 indicates that a certain portion of the previous memory should be forgotten, while a value of 1 indicates that that portion of the previous memory should be retained.

Input Gate: By adding the information that needs to be retained from the previous state to the information that needs to be remembered from the current state, we obtain the new memory state.

Output Gate: This gate integrates $c_{t}$ to produce an output.

The calculation formulas for each parameter in the structural diagram are shown in the following equations:

(32) $$\left\{\begin{array}{c} f_{t}=\sigma \left(W_{f}\cdot \left[h_{t-1},x_{t}\right]+b_{f}\right)\\ o_{t}=\sigma \left(W_{o}\cdot \left[h_{t-1},x_{t}\right]+b_{o}\right)\\ h_{t}=o_{t}*\tanh\left(C_{t}\right)\\ i_{t}=\sigma \left(W_{i}\cdot \left[h_{t-1},x_{t}\right]+b_{i}\right)\\ {\tilde{C}}_{t}=\tanh\left(W_{c}\cdot \left[h_{t-1},x_{t}\right]+b_{f}\right)\\ C_{t}=f_{t}*C_{t-1}+i_{t}*{\tilde{C}}_{t} \end{array}\right.$$

where $f_{t}$ is the output value of the forgetting gate; $i_{t}$ is the output value of the input gate; $o_{t}$ is the output value of the output gate; σ is the corresponding gate; and *W* is the corresponding parameter.

$c_{t}$ is the cell state (memory state), $x_{t}$ is the input information, and $h_{t-1}$ is the hidden state (derived from $c_{t}$).

BP (backpropagation) neural network is a multilayer neural network that uses error backpropagation. In systems such as signal processing and pattern recognition, multilayer feedforward networks are widely used models. However, most learning algorithms based on backpropagation for multilayer feedforward networks must rely on some form of nonlinear optimization techniques, which results in large computational costs and slow learning speeds. The theory of Radial Basis Function Neural Network provides a novel and effective means for learning in multilayer feedforward networks. RBF networks not only possess good generalization capabilities but also have lower computational requirements, with learning speeds generally much faster than other algorithms. The following diagram illustrates a simplified model of the RBF network.

**Figure 18:**
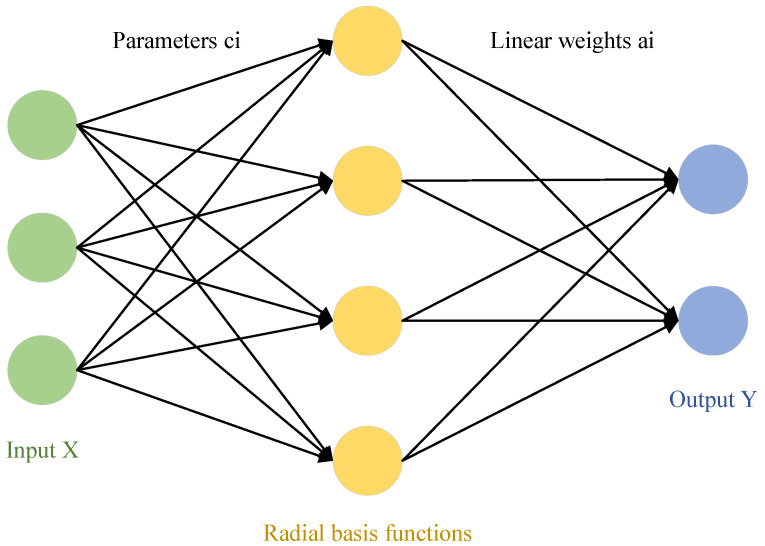
Simple radial basis function network structure.

After generalization, the applicability is significantly increased. The mapping from the input layer to the hidden layer (radial basis function layer) is a nonlinear mapping, with the basis function being the Gaussian function:

(33) $$R_{i}\left(x\right)=exp\left[-\frac{\|x-c_{i}{\|}^{2}}{2\sigma_{i}^{2}}\right]$$

where i=1,2,…,m; *x* is an n-dimensional input vector; $c_{i}$ is the center value of the *i*-th basis function, which has the same dimensionality as the input vector; $\sigma_{i}$ represents the normalization constant of the width of the *i*-th basis function’s center; and $\|x-c_{i}\|$ is the norm of the vector $x-c_{i}$, indicating the distance between *x* and $c_{i}$. The Gaussian function value $R_{i}\left(x\right)$ reaches its unique maximum at a specific center value of the basis function. According to the above function, as $\|x-c_{i}\|$ increases, the value of the basis function $R_{i}\left(x\right)$ decreases, approaching zero. For a given input value $x\in R^{n}$, only a small region near the center of *x* is activated, the radial basis function is shown in Figure 18.

To evaluate the computational complexity of the proposed method, a detailed analysis was conducted to estimate the number of floating-point operations required to compute the control law. The proposed control method consists mainly of the LSTM model and the MPC optimization problem. In the LSTM model, the computational complexity of state estimation is O(n^2^), where n is the number of neurons in the hidden layer, while during the prediction phase, the complexity is O(n), as only a forward pass is needed to compute the output. In contrast, the MPC controller involves solving an optimization problem, which typically has a complexity of O(n^3^), where N is the prediction horizon length. By integrating the LSTM and DDQN models into the MPC framework, the proposed method significantly reduces the computational load per control update, eliminating the need for solving large-scale optimization problems at each control step as required by traditional methods.

By comparing the computational complexity of the traditional MPC method with that of the proposed method, we estimate that the number of floating-point operations required for each control update is approximately X operations for the proposed method, compared to Y operations for the traditional MPC method. This demonstrates the clear computational efficiency advantage of the proposed method, particularly in real-time applications where computational burden is a critical factor.

## 5. Comparison of Simulation Results

From previous experiments on speed prediction, we understand that the prediction horizon is a crucial parameter. A horizon that is too short imposes a significant computational burden on the system, hindering the real-time update of vehicle information, while a horizon that is too long may result in poor prediction accuracy. Therefore, selecting an appropriate prediction horizon is vital for the experiments. In this paper, we base our predictions on LSTM and adjust the horizon to 5 s, 10 s, 15 s, and 20 s. In reference [40], it was pointed out that either too short or too long LSTM prediction field of view would have a negative impact on the experiment, so this paper chose the prediction field of view between 5 s and 20 s for testing.

**Figure 19:**
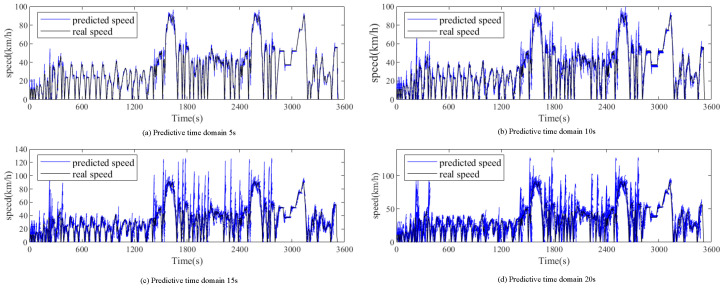
Prediction results of RBF with different horizons.

This study focuses on the LSTM-MPC-DDQN energy management strategy with a historical time domain of 40 s and prediction horizons of 5 s, 10 s, 15 s, and 20 s. The prediction results are shown in Figure 19. The experimental results are compared with two other control strategies. The cycling conditions for all control strategies are based on the cycling conditions processed in the previous chapters, with the initial SOC set to 90.

### 5.1. Training Process

**Figure 20:**
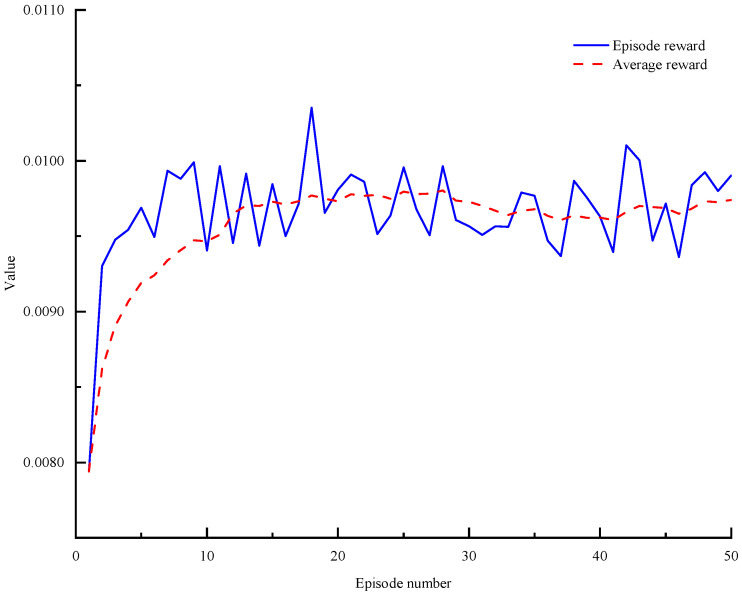
DDQN training flowchart.

The goal of deep reinforcement learning is to maximize rewards, and the effectiveness of DDQN training is measured by the increase in Q-values, which should ultimately stabilize and converge to a maximum value. Therefore, the choice of hyperparameters is crucial for the control effectiveness of deep reinforcement learning in energy management. The DDQN energy management system employs a three-layer BP neural network with 84 neurons in the hidden layer. In the velocity-prediction-based MPC-DDQN energy management, a four-layer neural network is used with 124 hidden neurons. The parameter update algorithm for all reinforcement learning neural networks utilizes the RMSProp algorithm, with a learning rate of 0.0001 and a sampling time of 0.1 s. Figure 20 illustrates the training process of the DDQN energy management agent.

As shown in Figure 20, with the increase in iterations, the reward value rises rapidly in the initial stages. However, after the 15th iteration, even though the number of iterations continues to increase, the reward value does not rise further but stabilizes around a certain value. At this point, the network essentially stops updating. This phenomenon is primarily due to the simplicity of the network and the limited input to the DDQN, resulting in a weak perception of the environment and, consequently, less information learned by the network. The velocity-prediction-based MPC-DDQN effectively addresses this issue. The velocity prediction model extracts historical velocity features by processing historical speed data, and the predicted speed information is input into the control strategy to calculate the predicted power. The DDQN then calculates the engine output power based on this predicted power. This method not only provides DDQN with more state information but also allows reinforcement learning to plan better over time, leading to the convergence of reward values while enhancing the vehicle’s economy.

**Figure 21:**
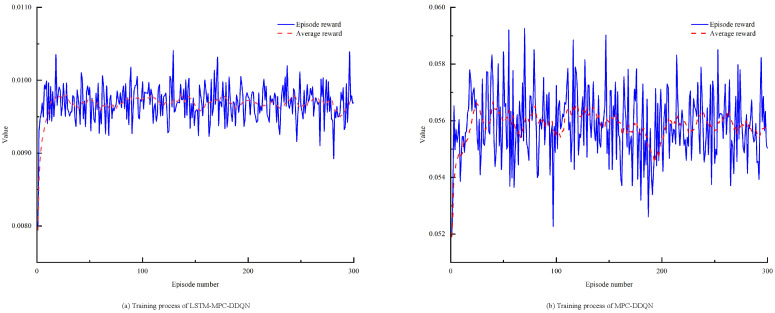
Training process of speed prediction energy management for different speed prediction methods.

The goal of deep reinforcement learning is to maximize rewards, and the effectiveness of DDQN training is assessed by the increase in Q-values, which should ultimately stabilize and converge to a maximum value. Therefore, the choice of hyperparameters is crucial for the control effectiveness of deep reinforcement learning in energy management. The DDQN energy management system employs a three-layer BP neural network with 64 hidden units. In the velocity-prediction-based MPC-DDQN energy management strategy, a four-layer neural network is used, also with 64 hidden units. The parameter update algorithm for all reinforcement learning neural networks utilizes the RMSProp algorithm, with a learning rate of 0.0001, a sampling time of 0.01 s, and an episode setting of 50. Figure 20 illustrates the training process of the DDQN energy management agent. Figure 21 further demonstrates the training process of the LSTM-MPC-DDQN energy management strategy, indicating that the prediction accuracy and stability of LSTM-MPC-DDQN are superior to those of the MPC-DDQN energy management strategy.

### 5.2. Simulation Experiment

As shown in Table 6, with the increase in the prediction horizon, both the average prediction time and RMSE also increase, indicating a decline in prediction accuracy and a reduction in stability.

**Figure 22:**
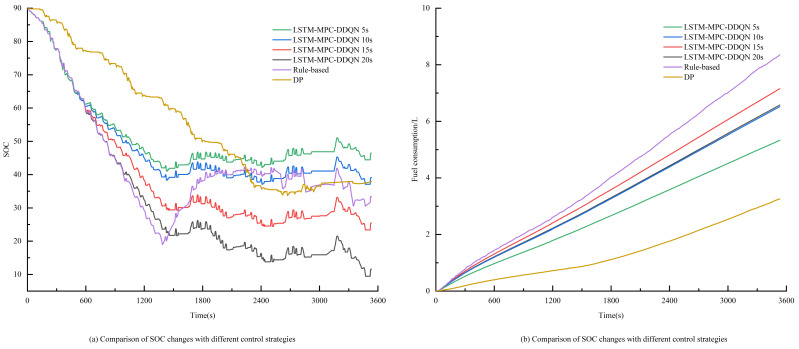
Variation curves of SOC and fuel consumption for three strategies.

As shown in Figure 22, the variations in SOC (state of charge) and fuel consumption under different control strategies are presented, with an initial SOC of 90% for all strategies. From Figure 22a, it can be observed that the DP and RB control strategies result in a final SOC close to 35%. However, when the prediction horizons for LSTM-MPC-DDQN are set to 15 s and 20 s, the final SOC values are both below 30%. This indicates that if the prediction horizon is set too long, it can lead to excessive discharge of the battery pack, coupled with a gradual decline in prediction accuracy.

Table 7 presents the simulation results for various control strategies, indicating that the fuel consumption of the dynamic programming (DP) approach is the lowest. The LSTM-MPC-DDQN energy management strategy, with a prediction horizon of 5 s, has equivalent fuel consumption closest to that of the dynamic programming algorithm. The dynamic programming algorithm is a global optimization strategy capable of finding the theoretical optimal solution and is commonly used as a reference standard for the control effectiveness of other strategies. To ensure a fair comparison, the objective function of the dynamic programming approach is the same as that of the proposed control strategies. The results show that, compared to the RB control strategy, the equivalent fuel consumption of the LSTM-MPC-DDQN strategy based on speed prediction is significantly reduced. This is because the RB strategy relies solely on predefined rules for power distribution, heavily depending on past experiences, and cannot adaptively adjust. Therefore, the control effectiveness of the RB strategy is generally not as effective as that of the LSTM-MPC-DDQN energy management strategy.

The dynamic programming algorithm is a global optimization strategy that can find the theoretical optimal solution and is commonly used as a reference standard for the control effectiveness of other strategies. To ensure a fair comparison, the objective function of dynamic programming is the same as that of the control strategies proposed in this study. The results indicate that, compared to traditional power tracking control strategies and the ECMS control strategy, the equivalent fuel consumption of speed prediction-based reinforcement learning energy management is significantly reduced.

**Figure 23:**
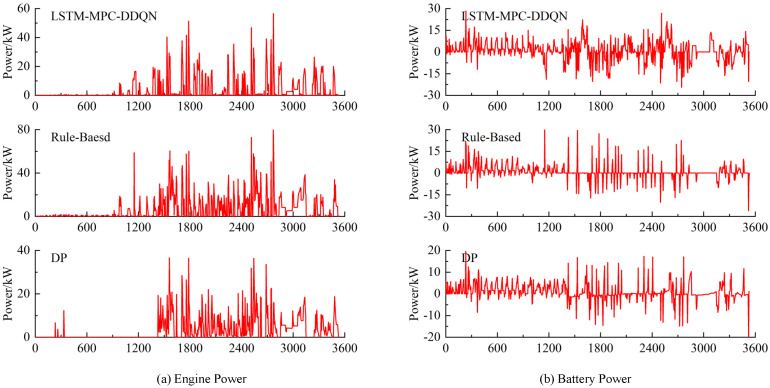
Variation of engine and battery pack output power under different control strategies.

As shown in Figure 23, the output power of the engine and battery pack under different control strategies reveals that the output power of the dynamic programming (DP) and LSTM-MPC-DDQN strategies is lower than that of the RB strategy. This is primarily because DP, as a global planning control strategy, can plan across a global range. Additionally, the LSTM-MPC-DDQN control strategy utilizes velocity prediction to make better judgments in the time domain, avoiding the operation of the engine in inefficient areas. In contrast, the RB strategy can only distribute power according to predefined rules, lacking adaptive regulation and flexibility. It frequently switches during operation and, compared to DP and LSTM-MPC-DDQN, utilizes the battery pack less often, resulting in higher fuel consumption. On the other hand, based on previous experiments, setting the prediction horizon of LSTM-MPC-DDQN to 5 s not only ensures fuel economy but also maintains performance.

**Figure 24:**
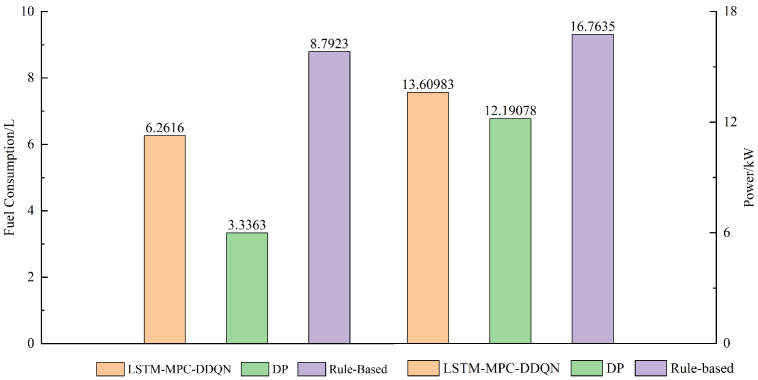
Variation in engine and battery pack output under different control strategies.

As shown in Figure 24, by integrating the fuel consumption and output power of each control strategy at every moment, we can obtain the total fuel consumption and total power output of the vehicle. Considering the fuel consumption data, the dynamic programming (DP) algorithm proves to be more efficient compared to the RB strategy proposed in this study. This is because both dynamic programming and LSTM-MPC-DDQN strategies can optimize planning in both the global and temporal domains, reducing unnecessary power output and fuel consumption, and thereby significantly enhancing overall efficiency. In contrast, the RB strategy, due to its inability to adapt to changing rules, fails to effectively predict and plan the power distribution between the engine and battery pack, resulting in relatively lower fuel efficiency.

**Figure 25:**
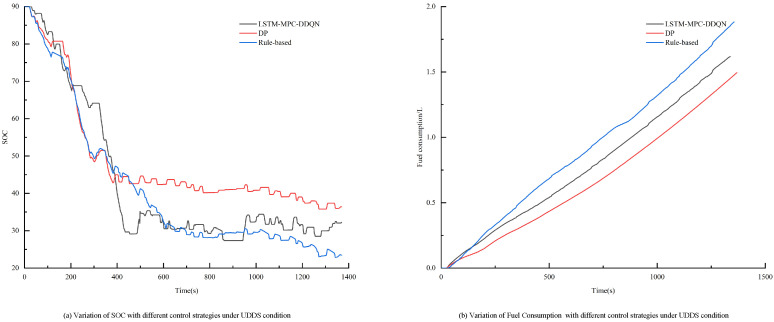
Variation in SOC and fuel consumption for different strategies under UDDS conditions.

To verify the effectiveness of the proposed control strategy, simulations were conducted under the UDDS conditions and compared with other control strategies. The simulation results are shown in Figure 25. The figure indicates that the final SOC of our proposed strategy approaches the target value of 30%. In contrast, the RB strategy consumes more energy and fuel. This observation is supported by the data in Table 8, which demonstrates that the fuel economy of the LSTM-MPC-DDQN control strategy is superior to that of the RB control strategy.

## 6. Conclusions

### 6.1. Contribution

In this study, we developed a P2 architecture model for PHEV, which includes a comprehensive vehicle powertrain system. This model enables the calculation of optimal operating trajectories for critical powertrain components, specifically the engine and battery pack. To address the limitations of traditional Dynamic Programmed Recognition (DPR) methods, which often fail to fully recognize specific drive modules, we proposed an innovative driving mode recognition system. This system employs a fuzzy controller to segment standard driving cycles into distinct modes, allowing for real-time driving mode identification. Additionally, we designed an LSTM-based speed prediction model, trained on a dataset incorporating widely adopted driving cycles such as FTP75, NEDC, and CBDC. To further refine this model, a DDQN algorithm was implemented to optimize the initial learning rate and dropout probability of the neural network. By integrating LSTM predictions within an MPC framework, we constructed an advanced LSTM-MPC-DDQN energy management controller. Simulation results demonstrate that this proposed strategy surpasses other energy management approaches in performance. Specifically, when compared to conventional RB control strategies, the LSTM-MPC-DDQN controller achieves significantly improved fuel efficiency, with fuel consumption levels approaching those obtained through the DP algorithm. Furthermore, the LSTM-MPC-DDQN strategy ensures stable SOC management, effectively maintaining SOC near its set target. The accuracy of speed predictions directly influences the power demand calculation within the prediction horizon, which in turn impacts the effectiveness of reinforcement learning for future power management planning. Finally, additional simulations using the UDDS cycle confirmed the strategy’s suitability for real-time applications, reinforcing its potential for practical implementation.

In the experiments conducted on the LSTM-MPC-DDQN energy management strategy, four prediction horizon lengths (5 s, 10 s, 15 s, and 20 s) were set. The results demonstrated that when the prediction horizon was 5 s, the strategy exhibited optimal performance, achieving the lowest equivalent fuel consumption at only 6.3220 L, representing a fuel saving of 2.034 L compared to the rule-based strategy. Additionally, the DP strategy achieved the lowest fuel consumption at 3.3856L. Due to its global optimization capabilities, the dynamic programming algorithm can identify the theoretical optimal solution and is commonly used as a benchmark for assessing the control effectiveness of other strategies. Verification under the UDDS driving cycle indicated that the LSTM-MPC-DDQN strategy saved 0.2729 L of fuel compared to the rule-based strategy and exhibited a fuel consumption difference of only 0.0749 L compared to the DP strategy.

This study proposes an innovative LSTM-MPC-DDQN energy management controller for PHEVs, which significantly improves fuel efficiency and stable SOC management compared to conventional strategies, as demonstrated through comprehensive simulations.

### 6.2. Outlook

In this study, all our experiments were conducted on the Simulink platform. Although Simulink allows for precise control of variables and exploration of various scenarios, it lacks many uncertainties present in real-world operations. Additionally, in our experiments, we did not consider the impact of slope on the controller. In the future, we plan to conduct real-vehicle tests to obtain real-world operational data, such as data from urban roads, highways, mountainous regions, and other complex environments.

## Figures and Tables

**Table 1:** Vehicle powertrain parameters.

Name	Parameters	Value	Parameters	Value
Vehicle	Total mass	1645 kg	Aerodynamic drag coefficient	0.3618
	Front area	2.11 m^2^		
Engine	Maximum speed	5000 rpm	Engine type	petrol engine
	Maximum torque	175 Nm		
Motor	Maximum speed	11,000 rpm	Motor type	permanent magnet synchronous
	Maximum torque	200 Nm		
Generator	Maximum speed	10,000 rpm		
	Maximum torque	180 Nm		
Battery	Voltage	650 V	Capacity	382 Ah

**Table 2:** Driving mode data.

Time (s)	Speed (km/h)
0	0
100	20
120	0
200	0
400	100
500	0

**Table 3:** MPC-DDQN energy management pseudo-code.

MPC-DDQN	
1	Define hyperparameters: greedy algorithm coefficient ϵ, size of replay buffer *D*, and discount factor γ.
2	Initialize the parameters of the current network ω and copy ω to the target network parameters $\omega^{-}$.
3	Set the maximum number of episodes $E_{m}ax$.
4	Loop through episodes for $i=1:E_{m}ax$.
5	Randomly initialize the state sequence *S* (predicted power demand sequence, SOC).
6	For each time step j=1:T
	Select an action *a* with probability ϵ or select an action $a^{*}$ with the maximum Q-value with probability (1−ϵ). Use the target network to compute the target value:
7	Compute the Temporal Difference (TD) error $\delta_{t}=y_{t}-Q\left(s_{t},a_{t},\omega \right)$.
8	Store the experience in the replay buffer using prioritized experience replay and sampling probabilities.
9	Update the current network’s parameters using samples from the replay buffer.
10	Every C step, copy the parameters of the current network ω to the target network $Q_{target}\left(S,a;\omega^{-}\right)$.
11	End of the time step.
12	End of the episode.
13	Take the first action from the optional output sequence as the range extender’s output power for the next time step, achieving continuous control via a rolling window.

**Table 4:** Speed interval division.

	$V_{a}$—Low	$V_{a}$—Middle	$V_{a}$—High
Mean velocity range	<15 km/h	15 km/h ⩽ $V_{a}$ < 25 km/h	⩾25 km/h
Maximum velocity range	<25 km/h	25 km/h ⩽ $V_{a}$ < 45 km/h	⩾45 km/h

**Table 5:** Fuzzy rules.

	$V_{a}$—Low	$V_{a}$—Middle	$V_{a}$—High
$V_{\max}$—Low	Low	Middle	∖
$V_{\max}$—Middle	Low	Middle	High
$V_{\max}$—High	Middle	Middle	High

**Table 6:** Performance LSTM vehicle speed prediction method.

Predictive Time	LSTM	
Domain	Average Prediction Time (s)	RMSE
5 s	0.001279	0.8344
10 s	0.005010	1.6456
15 s	0.009136	2.1546
20 s	0.014218	2.2019

**Table 7:** Simulation results of different control strategies.

Control Strategy	SOC Final Value/%	Fuel Consumption/L	Equivalent Fuel Consumption/L
LSTM-MPC-DDQN 5 s	0.4646	6.2616	6.3220
LSTM-MPC-DDQN 10 s	0.3912	6.5769	6.6278
LSTM-MPC-DDQN 15 s	0.2545	7.1513	7.1861
LSTM-MPC-DDQN 20 s	0.1160	6.5157	6.5308
Rule-based	0.3328	8.7923	8.8356
DP	0.3792	3.3363	3.3856

**Table 8:** Simulation results of different control strategies under UDDS condition.

Control Strategy	SOC Final Value/%	Fuel Consumption/L	Equivalent Fuel Consumption/L
LSTM-MPC-DDQN	0.3225	1.6185	1.7153
DP	0.3640	1.4948	1.6404
Rule-based	0.2345	1.8827	1.9882

## Data Availability

Data are contained within the article.
